# Genomic insights into the cellular specialization of predation in raptorial protists

**DOI:** 10.1186/s12915-024-01904-2

**Published:** 2024-05-07

**Authors:** Zaihan Li, Xiao Chen, Fangqing Zhao, Miao Miao

**Affiliations:** 1 University of Chinese Academy of Sciences, Beijing, 100049 China; 2 Laboratory of Marine Protozoan Biodiversity and Evolution, Marine College, Shandong University, Weihai, 264209 China; 3 Institute of Zoology, Beijing Institutes of Life Science, Chinese Academy of Sciences, Beijing, 100101 China

**Keywords:** Ciliates, Haptoria, Predation, Single-cell genomics, Toxicyst

## Abstract

**Background:**

Predation is a fundamental mechanism for organisms to acquire energy, and various species have evolved diverse tools to enhance their hunting abilities. Among protozoan predators, raptorial Haptorian ciliates are particularly fascinating as they possess offensive extrusomes known as toxicysts, which are rapidly discharged upon prey contact. However, our understanding of the genetic processes and specific toxins involved in toxicyst formation and discharge is still limited.

**Results:**

In this study, we investigated the predation strategies and subcellular structures of seven Haptoria ciliate species and obtained their genome sequences using single-cell sequencing technology. Comparative genomic analysis revealed distinct gene duplications related to membrane transport proteins and hydrolytic enzymes in Haptoria, which play a crucial role in the production and discharge of toxicysts. Transcriptomic analysis further confirmed the abundant expression of genes related to membrane transporters and cellular toxins in Haptoria compared to Trichostomatia. Notably, polyketide synthases (PKS) and l-amino acid oxidases (LAAO) were identified as potentially toxin genes that underwent extensive duplication events in Haptoria.

**Conclusions:**

Our results shed light on the evolutionary and genomic adaptations of Haptorian ciliates for their predation strategies in evolution and provide insights into their toxic mechanisms.

**Supplementary Information:**

The online version contains supplementary material available at 10.1186/s12915-024-01904-2.

## Background

Predation plays a crucial role as a fundamental mechanism by which organisms acquire the necessary energy for survival and growth. Through the active pursuit and capture of prey, animals engage in dynamic predator-prey relationships that shape ecosystems and drive the intricate web of life. In their pursuit of sustenance, organisms have evolved a remarkable assortment of tools to enhance their hunting capabilities. For instance, *Rana pipiens* employs a viscoelastic tongue to efficiently capture crickets [1], while *Naja pallida* immobilizes mice with its venomous fangs [2, 3]. These diverse tools exemplify the distinct adaptations possessed by different species to facilitate their hunting activities. Each species possesses specialized instruments uniquely suited to its ecological niche and prey preferences. Furthermore, as our knowledge of the microscopic world deepens, it has become apparent that eukaryotic microbes, often regarded as the foundation of the food chain, have also developed their own predatory weapons [4, 5]. Protists, as the deepest branches on the eukaryotic tree [6], also possess a vast genetic diversity within the eukaryotic organisms [7, 8]. These single-cell organisms serve as the ancestral forms for many functions that are essential in higher organisms. For example, micropinocytosis, essential for mammals, can be traced to at least the shared ancestor of Amoebozoa and Opisthokonts [9, 10]. Among the diverse groups of protists, ciliates are widely distributed in various aquatic environments, standing out as particularly intriguing [11, 12]. Ciliates exhibit remarkable functional characteristics that contribute to their enhanced survival. For example, *Spirostomum* shows an ability for responsive contraction movements through its mesh-like contractile fibrillar system [13, 14]. In contrast, plagiopylean ciliates acquire energy in anaerobic environments by forming endosymbiotic relationships with anaerobic prokaryotes like archaeal methanogens and denitrifying bacteria [15–17].

Extrusomes are cellular organelles that play a significant role in predator-prey interactions among ciliates. For ciliates that with the most in-depth study, such as *Tetrahymena*, *Paramecium*, and *Frontonia*, these specialized cellular structures serve as defense mechanisms against predator attacks [18, 19]. When a potential threat makes contact with *Paramecium*, it induces a rapid and explosive extrusion of trichocysts, enabling the ciliate to swiftly escape. Mucocysts also appear in prey ciliates such as *Tetrahymena*, functioning to respond by releasing compounds that neutralize the attack [20]. However, there is an exception to this pattern observed in Haptoria. As natural predators, Haptoria have evolved offensive extrusomes, known as toxicysts [21–24]. These toxicysts, located in their oral apparatus, are designed for predation and are rapidly discharged upon contact with prey, immobilizing them. Wessenberg and Antipa [25] discovered that when the proboscis of *Didinium* contacts with *Paramecium*, elongated extrusive organelles are instantaneously released from the oral apparatus, facilitating the capture and killing of the prey [24, 26]. The toxicysts in their resting state exhibit a highly intricate and delicate structure, consisting of an external capsule covered by a thick wall and an internal tube. It is believed that unknown toxins accumulate within the tube and are released into the prey’s body through the telescopic discharge of the tubule and/or the fusion of the toxicyst membrane with the plasma membrane [27]. Toxicysts are not exclusive to haptorian ciliates. For example, *Coleps* from the class Prostomatea also possesses these toxic structures [28]. Trichostomatia, commonly found in the rumen of herbivorous mammals, has been confirmed through electron microscopy to lack the characteristic extrusome structure of Haptoria [29].

Currently, ongoing studies are dedicated to exploring the structure and discharge mechanism of toxicysts in Haptorian ciliates. This aspect has been more extensively studied in *Coleps*, where the discharge of toxicysts was found to be linked with the significant production of free fatty acids (FFAs), as revealed by mass spectrometry techniques [28]. However, our understanding of the genetic processes responsible for toxin production and the specific types of toxins contained within toxicysts remains limited. To address this gap, we utilized single-cell sequencing technology to obtain genome sequences from seven species of Haptorian ciliates collected from both marine and freshwater environments. Through comparative genomic analysis, we successfully identified mechanisms influencing the discharge of toxicysts. This research sheds light on how these haptorian protists employ tools in their predation activities.

## Results

### Morphology and lifestyle

**Fig. 1:**
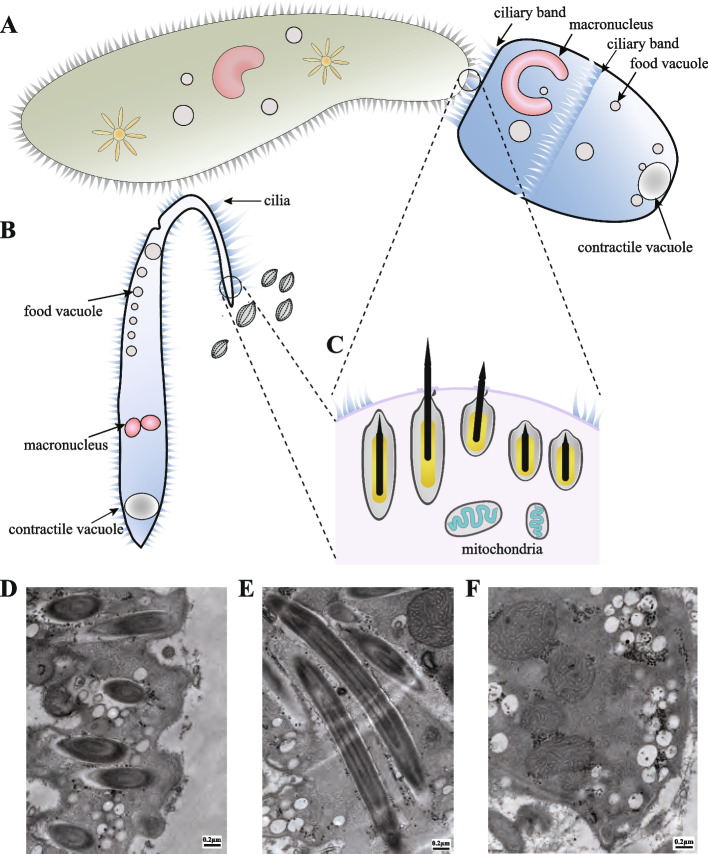
Morphology of Haptoria and their subcellular structures. **A** *Didinium nasutum* (blue on the right) preying on *Paramecium caudatum* (green on the left), with *Didinium* utilizing toxicysts near its proboscis to immobilize its prey. **B** *Dileptus* sp. (blue on the left) preying on *Tetrahymena* (gray on the right). **C** Internal view of toxicysts within the surface of a Haptoria ciliate, displaying a toxicyst in the process of discharging. **D**-**F** Subcellular structures in a raptorial ciliate, *Litonotus* sp., using transmission electron microscope. **D** Short type II toxicysts. **E** Long type I toxicysts. **F** Mitochondria

To investigate the specific mechanisms of raptorial ciliates, we collected a total of seven Haptorian ciliates, including two species from seawater and five from freshwater. Despite their large size and comparatively limited maneuverability compared to their prey, these Haptorian ciliates exhibit remarkable predation abilities across diverse aquatic environments. For example, the oval-shaped *Didinium* mainly engulfs *Paramecium*, whereas *Dileptus*, possessing a slender form, consumes *Tetrahymena* (Fig. 1A, B). Despite variations in predation strategies and morphological characteristics, Haptoria species are adept at preying on medium-sized protozoa, utilizing their toxicysts as their predatory instruments.

It is believed that subcellular structures play an important role in the distinct predatory strategies of ciliates through the development of specialized organelles, such as proboscis and extrusomes [22, 24, 30]. To examine the intricate architecture of ciliates, we used a transmission electron microscope (TEM) to investigate the subcellular structures in *Litonotus*. TEM enabled us to highlight the organelles involved in the raptorial lifestyle. Notably, Fig. 1D, E presents TEM images depicting the different types of toxicysts identified in *Litonotus*, which have been classified as long toxicysts and short pexicysts based on their distinctive morphological characteristics, as observed in *Didinium nasutum* [25]. We further provided a detailed examination of the toxicyst region (Fig. 1C), which typically serves as the initial point of contact between the cell and its prey during the raptorial feeding process. Several Haptoria species possess two types of toxicysts with varying sizes and structures. Specifically, type I toxicysts commonly appear as elongated rod-like shapes and are consistently present across all Haptoria species, whereas type II toxicysts (pexicysts) are slightly shorter and may be absent in certain species [27].

### Genome assembly of Haptorian ciliates

To unravel the functional role of toxicysts, we performed a comprehensive analysis of genomic data to identify the genes associated with their formation. After eliminating contamination originating from bacteria and algae, 7 ciliate genomes show different N50 lengths (1653 ~ 16,689 bp) and the largest contig length (38,189 ~ 287,940 bp). The average size of the seven genome assemblies was found to be 72 Mb, with GC contents ranging from 22.59 to 35.86%. Through a combination of ab initio, homology-based, and transcriptome-guided gene prediction, we finally obtained 18,691 ~ 32,428 protein-coding genes for each species (Table 1), comparable to other Litostomatea species, particularly those belonging to the Haptoria group. The completeness of these assembled genomes was evaluated using EukCC [31], with the majority of them achieving over 85% completeness, except for *Trachelophyllidae* spp., which exhibited a completeness level of 66.7%.

**Table 1:** Single-cell genome assemblies of Haptoria

**Species**	**Clean reads (Gb)**	**Genome Size (bp)**	**Largest contig (bp)**	**N50 (bp)**	**GC (%)**	**Genome completeness (%)**	**No. of genes**
*Lacrymaria* sp.1	20.5	99,803,170	137,119	2832	33.6	97.0	32,428
*Lacrymaria* sp.2	19.9	49,422,897	53,718	3007	29.0	90.9	22,896
*Didinium* sp.1	72.0	72,122,335	137,092	10,023	24.9	93.9	21,837
*Trachelophyllidae* spp.	40.5	47,450,859	101,362	1146	36.2	66.7	20,269
*Monodinium* sp.	30.6	45,171,837	38,189	1889	31.7	87.9	18,691
*Litonotus* sp.1	19.4	39,834,529	71,383	1993	26.2	93.9	17,390
*Dileptus* sp.	58.7	96,500,208	53,007	2421	25.7	90.9	26,891

### Comparative genomics and phylogenomic analysis

**Fig. 2:**
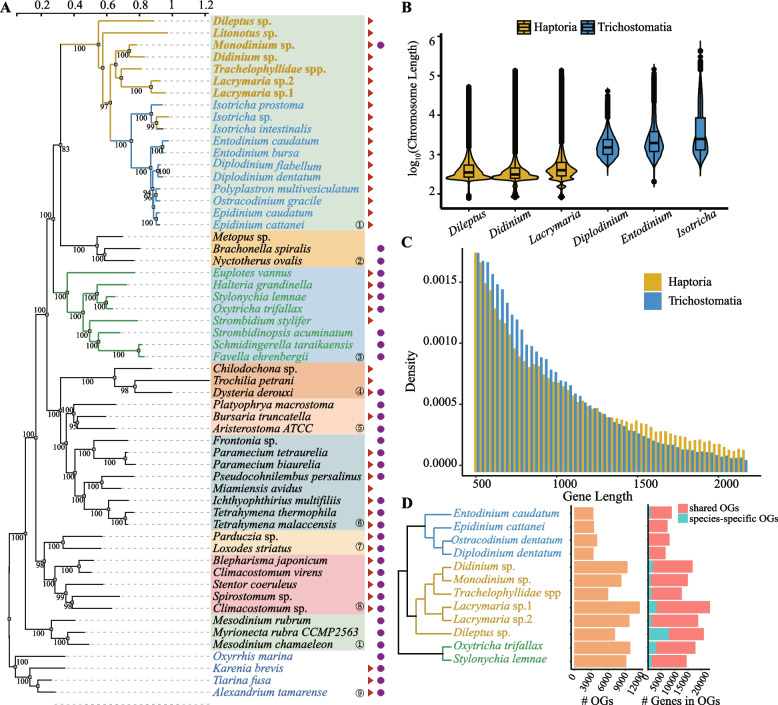
Phylogenomic analyses of Litostomatea with other ciliates. **A** Phylogenomic tree of ciliates constructed using ML, depicting the evolutionary relationships among different ciliate classes. The numbered labels represent the following classes: ① Litostomatea (subclass Haptoria in yellow and subclass Trichostomatia in blue), ② Armophorea, ③ Spirotrichea, ④ Phyllopharyngea, ⑤ Colpodea, ⑥ Oligohymenophorea, ⑦ Karyorelictea, and ⑧: Heterotrichea. Species with red triangles indicate the availability of genome data, and purple dots indicate the availability of transcriptome data. **B**, **C** Statistics on the assembly and annotation of Haptoria and Trichostomatia genomes, showing the distribution of chromosomes and gene length. **D** Comparision of orthologs and gene counts in orthogroups among Haptoria, Trichostomatia, and Spirotrichea

To investigate the phylogenetic relationship of Haptoria within the Ciliophora, we conducted an analysis encompassing a total of 57 species, including 53 ciliates and 4 apicomplexans. Among the 53 ciliates, 21 species belonged to the class Litostomatea. All other genomic or transcriptomic data utilized in this study were obtained from the SRA or Genome database in NCBI. As shown in Fig. 2A, the class Litostomatea could be classified into 3 distinct clades. The order Cyclotrichida, consisting of a sole family *Mesodiniidae*/*Myrionecta*, appears isolated from the rest of the Litostomatea. This observation aligns well with the fact that Mesodiniidae exhibit a distinct lifestyle divergent from other organisms. Subsequently, we focused on the other two clades within the Litostomatea, which includes the 7 ciliates we sequenced. We compared the genome assemblies and gene predictions between Haptoria clade and Trichostomatia clade. We found that Haptoria exhibits a shorter average chromosome length compared to Trichostomatia (Fig. 2B). Furthermore, we observed notable differences in gene length between the 2 subgroups. Trichostomatia displayed a higher proportion of genes below 1000 bp, whereas Haptoria exhibited a higher proportion of genes exceeding 1300 bp (Fig. 2C). We also predicted genes in Mesodiniidae using the transcriptome data. The gene length distribution of Mesodiniidae is closer to the Trichostomatia clade, significantly differing from that of Haptoria (Additional file 1: Fig. S1).

**Fig. 3:**
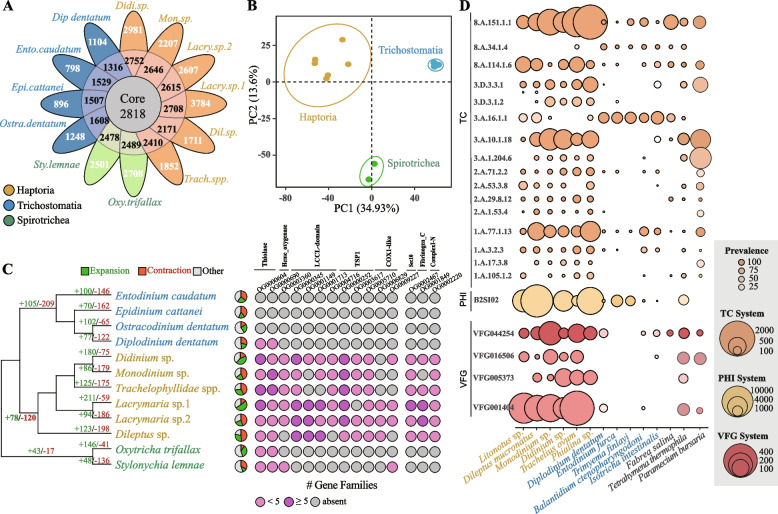
Comparative genomic analysis between Haptoria and Trichostomatia. **A** The Venn diagram illustrates the number of shared orthogroups among ciliates. The value inside the circles represent the number of core orthogroups shared by at least seven species, while the values outside the circles represent the number of species-specific orthogroups. **B** Principal component analysis of ciliate orthologs shows a clear segregation between Haptoria, Trichostomatia, and Spirotrichea. **C** Gene expansion and contraction analysis in Haptoria and related species (left). The expanded gene families are shown on the right panel. **D** Gene expression and prevalence analysis of TCDB, PHI-base, and VFDB genes in Haptoria, Trichostomatia, *Fabrea salina*, *Tetrahymena thermophila*, and *Paramecium bursaria*

To investigate the gene content within Litostomatea, we employed OrthoFinder [32] to compare the homologous genes between Haptoria and its closely related Trichostomatia, with 2 Spirotrichea species serving as outgroups. A total of 170,280 genes were clustered into 26,848 gene families across the 12 species, with 80.6% of these genes present in the analyzed orthogroups. The resulting clustered tree (Fig. 3C) generated from multiple sequence alignments shows a similar structure to the maximum likelihood (ML) tree in Fig. 2A. Notably, when examining the number of homologous genes in different species, a significant distinction between Haptoria and other species became apparent regardless of the gene count within each homologous group (Fig. 2D). From the perspective of orthogroups and gene numbers, Haptoria possesses a greater number of shared homologous genes than other members of the Litostomatea group.

In addition, we identified a specific set of 2818 orthogroups classified as core orthogroups, which were found in at least 6 species (Fig. 3A). Haptoria species exhibited a significantly higher number of core orthogroups, with a mean value of 2550, nearly double that of Trichostomatia (1490). To further assess the relationship among these predatory ciliates, we employed principal component analysis (PCA) to assess the differences between Haptoria, Trichostomatia, and Spirotrichea. As a result, more evidence suggests that the ortholog profiles in Haptoria can also be significantly distinguished from other families (Fig. 3B).

### Toxic-related and transmembrane gene expansions in Haptoria

To explore the factors contributing to the genomic divergence within Litostomatea, we focused on the gene expansion events in Haptoria. To determine the divergence time of Litostomatea, we used both the BEAST and R8S [33] methods, employing Spirotrichea as a temporal reference point due to their inclusion in the fossil record [34]. The ultrametric tree generated by R8S is more similar to the phylogenetic tree, and thus, we chose the analysis results of R8S. We identified 78 expanded gene families and 120 contracted gene families in the Haptoria clade. Further examination of the expanded gene families using the PFAM database [35] allowed us to identify eight gene families: Thiolase, Heme_oxygenase, Sec10 Exocyst, LCCL-domain, ComplexI-N, Fibrinogen_C, TSP1, and COX1-like (Additional file 1: Table S1). These gene families, associated with membrane transport proteins and hydrolytic enzymes, exhibited higher abundance within the Haptoria clade, while being largely absent in both Trichostomatia clade and Spirotrichea clade (Fig. 3C).

**Fig. 4:**
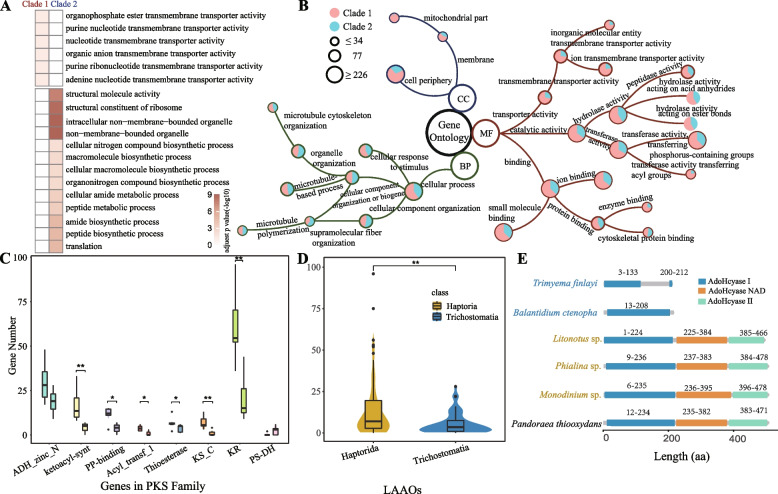
Functional enrichment analysis reveals *toxin-related gene expansions in Haptoria*. **A** Gene Ontology (GO) enrichment analysis of genes with increased copy numbers in clade 1 Haptoria and clade 2 Trichostomatia. **B** Gene Ontology (GO) enrichment analysis based on genes with increased copy numbers in clade 1 Haptoria and clade 2 Trichostomatia. The enriched categories include biological process (BP), cellular component (CC), and molecular function (MF). The red and cyan circles represent the proportions of gene copies from Haptoria and Trichostomatia, respectively. The size of the circles corresponds to the total number of gene copies. **C** Comparison of the eight conserved structural domains of the PKS (polyketide synthase) family between Haptoria and Trichostomatia from the Litostomatea class. The left panel shows the genes from Haptoria, while the right panel shows the genes from Trichostomatia. **p-value < 0.01 and *p-value < 0.05 by Student’s *t* test. **D** Comparison of the number of LAAO (l-amino acid oxidase) genes from the Litostomatea class between Haptoria and Trichostomatia. **p-value < 0.01 by Student’s *t* test. **E** Comparison of the AdoHcyase family in different ciliates with the bacterium *Pandoraea thiooxydans*

To investigate the toxicyst’s related membrane transport proteins and hydrolytic enzymes, we conducted a comprehensive analysis of the transcriptomic data from six Haptoria species and five Trichostomatia species, with *Tetrahymena thermophila*, *Paramecium bursaria*, and *Fabrea salina* as the outgroup species. We evaluated the expression of specific genes by comparing them to the TCDB [36], PHI-base [37], and VFDB [38] databases, and revealed a significantly higher abundance of membrane transport proteins and cellular toxins within Haptoria compared to both Trichostomatia and outgroup species (Fig. 3D, Additional file 1: Table S2). We further compared the orthogroup genes between Haptoria and Trichostomatia and found that genes from Haptoria were enriched in transmembrane transporter-related gene families, while Trichostomatia exhibited enrichment in biosynthetic and non-membrane-bounded genes (Fig. 4A). Additionally, we performed gene enrichment analysis for Haptoria using WEGO2.0 [39], which revealed significant enrichment in pathways related to microtubules, transmembrane transporters, and hydrolase activity (Fig. 4B).

To identify potential toxin genes in Haptoria, we leveraged toxin proteins available in the Uniprot database. Among the discovered genes, polyketide synthases (PKS) and l-amino acid oxidases (LAAO) stood out due to their extensive duplication events. PKSs play a crucial role in the biosynthesis of diverse secondary metabolites, including toxins, and exhibit eight conserved structural domains. [40] Interestingly, our analysis revealed a noteworthy disparity in the abundance of these domains between Haptoria and Trichostomatia genomes, with six domains being significantly more prevalent in Haptoria (Fig. 4C). Additionally, we explored LAAOs, enzymes responsible for the oxidative deamination of amino acids, and found that a unique structural domain exhibited a higher number of repeats in Haptoria genomes (Fig. 4D). This finding suggests a potential functional significance of LAAO in Haptoria’s predatory activities.

## Discussion

While the presence of toxicysts in Haptoria has long been acknowledged, the genetic makeup of this unique class has not been thoroughly investigated. One of the major difficulties in studying this group has been the inherent challenge of culturing these predators in large numbers within laboratory settings. Previously, genome sequencing efforts were primarily focused on parasitic ciliates within the Litostomatea class. However, by employing single-cell sequencing methods, we have been able to delve into the intricate genetic details using a limited number of ciliate cells. To address uncertainties regarding the termination codons of Litostomatea and the lack of protein evidence, we optimized the methods for genome assembly and gene prediction and effectively eliminated bacterial contamination. Consequently, we have successfully sequenced and assembled the single-cell macronuclear genomes of seven Haptorian ciliates.

In spite of their close evolutionary relationship, significant divergence was observed between Haptoria and other Litostomatea species. Firstly, our comparative genomics analysis provides support for the clear separation of Mesodiniidae species from other core species within the Litostomatea class. This finding indicates that the symbiotic family Mesodiniidae exhibits a significantly distinct genetic profile when compared to their predatory counterparts. Secondly, through the comparison of chromosome length and gene length, we identified notable distinctions between Haptoria and Trichostomatia. This observation is further reinforced by the analysis of orthologous genes between Haptoria and Trichostomatia. Compared to Haptoria, Trichostomatia exhibits a lower abundance of orthologous genes and genes within orthologous groups. Furthermore, the non-toxicyst clades (Trichostomatia and Spirotrichea) are characterized by a deficiency in gene families associated with enzyme activity and ionic transmembrane functions, whereas Haptoria shows a significant increase in the number of gene duplications associated with these functions. The majority of these gene families are associated with membrane transport proteins and hydrolytic enzymes, potentially playing a role in the predation mechanism of Haptorian ciliates. During the discharge of toxicysts, the fusion between the toxicyst membrane and the cell membrane has been observed. Previous studies have shown that this process is correlated with fluctuations in Ca^2+^ concentration, leading to the rapid expulsion of toxicysts from the cell [19, 23]. Transcriptome data further strengthens these findings by showing that ciliates from the Haptorian ciliates express a higher number of genes associated with membrane transport proteins and cellular toxins compared to other ciliate groups. AdoHcyase (S-adenosylhomocysteine hydrolase) family was screened from Haptoria transcriptomes as an essential hydrolase [41]. In Haptorian ciliates, members of this hydrolytic enzyme family exhibit generally higher gene expression levels and comparatively more intact gene structural domains. Conversely, within Trichostomatia, the same gene structure of this category appears to be absent (Fig. 4E). Furthermore, the presence of acid phosphatase, typically located in the lysosomes of animal cells, within Haptoria [26] suggests that these ciliates may employ a sophisticated set of hydrolytic enzymes to initiate prey digestion. It is worth noting that several extrusomes in protists, such as mucocysts in *Tetrahymena*, trichocysts in *Paramecium* (considered homologous organelles with mucocysts [42]), and rhoptries in Apicomplexans, are regarded as lysosome-related organelles [19, 43].

In terms of phenotype, the Litostomatea class can be divided into three distinct groups based on their feeding patterns: Mesodiniidae, known for their mixotrophic lifestyle with algae [44, 45]; Trichostomatia, which parasitize the rumen of mammals [46]; and Haptoria, characterized as free-living raptorial ciliates [47]. The diverse lifestyles within Litostomatea have resulted in significant evolutionary divergence. As parasitic species, Trichostomatia have undergone gene loss of many essential genes typically found in free-living ciliates. Instead, they have evolved CAZymes, similar to those found in gut microbes, to enhance the digestion of polysaccharides [46]. In contrast, the expansive aquatic environments in which Haptoria reside have imposed distinct evolutionary pressures, leading to the development of toxicysts as a crucial part of their predatory strategy. Compared to Trichostomatia, Haptoria exhibits a higher abundance and diversity of genes associated with transmembrane transport and cellular toxins within their genomes. This indicates that Haptoria have undergone specific adaptations, resulting in genes specialized for the discharge of toxicysts and the paralysis of prey.

In addition, our investigation revealed an increased number of gene copies in Haptoria, specifically within certain gene families. Notably, the PKS gene family, known for its gene duplications in toxin production in ciguatoxic dinoflagellates [48], exhibits a higher frequency of gene duplications in the genomes of Haptoria compared to other ciliates. Similarly, the LAAO gene, which is widely distributed in the venom glands of animals such as rabbitfish and pit vipers [49], is found in a higher number of copies in Haptoria. Previous studies have demonstrated that LAAO derived from the serum of *Siganus oramin* induces lysis and mortality in the parasitic ciliate *Ichthyophthirius multifiliis* [50]. Further studies are still needed to investigate the function of these genes on mediating the production and discharge of toxicysts in Haptorian ciliates.

## Conclusions

Considering that most of raptorial protists cannot be cultured in laboratories, by leveraging single-cell genome sequencing methods, we have been able to delve into the intricate genetic details of these predators using a limited number of ciliate cells. We have successfully sequenced and assembled the genomes of seven Haptoria ciliates. Comparative genomic analysis revealed distinct gene duplications related to membrane transport proteins and hydrolytic enzymes in Haptoria, which play a crucial role in the production and discharge of toxicysts. Transcriptomic analysis further confirmed the abundant expression of genes related to membrane transporters and cellular toxins in Haptoria compared to Trichostomatia. Notably, PKS and LAAO were identified as potentially toxic genes that underwent extensive duplication events in Haptoria.

## Methods

### Sample collection

Two species of *Lacrymaria* were isolated from seawater in Pier-bridge, Qingdao, China (36° 06′N, 120° 31′E). *Didinium* sp.1 was collected from the freshwater pond in Baihuayuan Park, Qingdao, China (36° 04′N, 120° 22′E). Trachelophyllidae spp. and *Monodinium* sp. were collected from the freshwater of Weishan Lake, Shandong, China (34° 67′N, 117° 24′E). *Dileptus* sp. and *Litonotus* sp. were collected from the freshwater of Jiudu River, Beijing, China (40° 37′N, 116° 38′E).

### Transmission electron microscope (TEM)

*Litonotus* was used for investigating subcellular structures in raptorial ciliates. For convenient observation, each *Litonotus* undergoes two to three washes with freshwater using glass pipettes. The samples were fixed using 2.5% glutaraldehyde (pH 7.4) for 24 h at 4 °C. Subsequently, the specimens were embedded in Eponate 812, followed by sectioning into ultrathin slices. To facilitate observation and photography using Tecnai 20 (FEI, Lausanne, Netherlands) electron microscopy, the sections were stained with uranyl acetate and lead citrate. The shape of extrusomes and spatial distribution of mitochondria is plural based on TEM.

### High-throughput sequencing and genome assembly

After conducting morphological observations and identifications, we individually aspirated ciliates that had undergone 12 h of starvation using glass pipettes, placed them in freshwater, and repeated this process three to four times to minimize bacterial contamination as much as possible, and macronuclear genomic DNA was amplified using REPLI-g Single Cell Kit (Qiagen). The Truseq Nano DNA HT Sample Preparation Kit (Illumina USA) was used for the preparation of sequencing libraries. High-throughput sequencing was performed on an Illumina HiSeq platform using 150 bp paired-end reads.

To assess the quality of the paired-end sequencing data, FastQC v0.11.9 [51] was employed. Subsequently, low-quality reads were filtered out using Trimmomatic v0.39 (HEADCROP:10 ILLUMINACLIP:2:30:10 SLIDINGWINDOW:4:19) [52]. The genome of seven species were assembled using SPAdes v3.13.0 with kmer options (-k 61, 71, 77, 81) [40]. To eliminate contamination resulting from bacteria and algae, assembled sequences were aligned to the NR databases using DIAMOND v0.9.26 (--db nr --outfmt 6 --evalue 1e-5 --max-hsps 1) [53] and MetaGeneMark v3.38 [54], and those assigned to bacteria or algae were identified and removed as contaminants.

Other 37 genomes datasets were collected from other ciliates classes retrieved from NCBI. All these genomic data were assembled using Spades v3.13.0(-k 21,33,55,77). Gene prediction was performed using AUGUSTUS v3.3.0. All the data sources are documented in Additional file 1: Table S3.

### Gene prediction and annotation

To enhance the accuracy of gene prediction in the assembled genomes, the following procedures were implemented: (1) AUGUSTUS v3.3.3 [55] was used to predict genes in the seven genome datasets obtained during this study, as well as all transcript data available in the NCBI database (Additional file 1: Table S3). The model was trained using the RNA-seq data of *Monodinium* sp. (2) Blast v2.10.0 was used to search for gene fragments that corresponded to transcripts in other members of the Litostomatea class. By combining de novo approaches with transcripts from related species, it was possible to identify species-specific genes within the Litostomatea class. (3) DIAMOND v0.9.26.127 was used to annotate protein-coding genes in the seven ciliate genomes by comparing against the NR database. EukCC v0.2 was used to assess the completeness of the genomes.

### Phylogenetic and gene family analysis

We collected the genomic or transcriptomic data of additional 53 ciliates and 5 apicomplexans to serve as outgroup taxa. GPSit (-b BMGE-1.12 -e 1e-5) [56] was used to align them with 157 ciliate orthologous genes. ML analysis was performed using IQ-Tree v1.6.12 [57] with the model LG + F + R6, and the results were evaluated using 1000 bootstrap replicates.

OrthoFinder v2.5.4 was applied to identify orthologous groups from the all-against-all comparisons based on DIAMOND. *Entodinium caudatum*, *Epidinium cattanei*, *Ostracodinium dentatum*, and *Diplodinium dentatum* were categorized as Trichostomatia, while *Oxytricha trifallax* and *Stylonychia lemnae* were categorized as Spirotrichea. Species divergence time was estimated using both BEAST v 1.10.4 (mcmc 1000000) and R8S v1.81 (nsites = 419706 age = 368) with the divergence time of *Entodinium* and *Dileptus* as the calibration point. Based on the gene family clustering and divergence time estimation, CAFÉ v4.2.1 [58] was used to identify the expansion and contraction events of gene families. Subsequently, to investigate the functional implications of these gene family expansions or contractions, we mapped the genes within these families to the PFAM database using InterProScan. We performed an analysis to identify significantly overrepresented Gene Ontology (GO) terms among the rapidly expanded gene families using the clusterProfiler package [59] and WEGO 2.0. This approach allows us to gain insights into the functional enrichment of these gene families in Haptorian ciliates.

### Transcriptome data analysis

All transcriptome data were sourced exclusively from the NCBI Short Read Archive (SRA). *Diplodinium dentatum*, *Entodinium furca*, *Trimyema finlayi*, *Balantidium ctenopharyngodoni*, and *Isotricha intestinalis* are representatives of the Trichostomatia. Transcripts from *Litonotus* sp., *Dileptus mucronatus*, *Monodinium* sp., *Didinium* sp.2, *Trachelius ovum*, and *Phialina* sp. were chosen as representatives of the Haptoria. We also collected other 29 transcriptomes from other species in ciliates (Additional file 1: Table S3). All the RNA-seq data were assembled using Trinity 2.8.5 [60]. *Fabrea salina* served as an outgroup. We employed the databases of TCDB, PHI-base, and VFDB to identify genes related to membrane transport and toxicity genes in these transcriptome data. The significance of the differences between expression abundance values was examined by Student’s *t* test with the significance threshold set at *p* < 0.01.

### Supplementary Information

Additional file 1: **Table S1. **Differential Gene Statistics of Orthogroups in Haptoria, Trichostomatia and Spirotrichea. Gene counts of orthogroups in eight expanded gene families among Haptoria, Trichostomatia and Spirotrichea. Did.sp., Dilep.sp., Lacry.sp.1, Lacry.sp.2, Mono.sp., Trach.spp. from Haptoria, Ostra.dentatum, Dip.dentatum, Ento.caudatum, Epi.cattanei from Trichostomatia and Oxy.trifallax, Sty.lemnae from Spirotrichea. **Table S2. **DE genes associated with TCDB, PHI, VFDB in Haptoria, Trichostomatia and three Outgroup species. Differentially expressed gene about membrane transport proteins and cellular toxins. Lit.sp., Dil.mucronatus, Mon.sp., Did.sp., Tra.ovum, Phi.sp from Haptoria, Dip.dentatum, Ento.furca, Tri.finlayi, Bal.ctenopharyngodoni, Iso.intestinalis from Trichostomatia and Fab.salina, Tetra.thermophila, Param.bursaria as Outgroup species. **Table S3. **The source of ciliates genome and transcriptome data. We demonstrate the sources of all 62 genomic and transcriptomic datasets utilized in this study. **Fig. S1.** The distribution of gene length in litostomatea. Haptoria, Trichostomatia and Mesodinium, representing three types in litostomatea are all included.

## Data Availability

All the data supporting the findings of this study are available and included in this published article, its supplementary information file (Additional file 1: Table S3), and publicly available repositories. Genomic data generated in this study have been deposited to the NGDC database (https://ngdc.cncb.ac.cn/gsa/) under BioProject number PRJCA019558.

## Abbreviations

PKS: Polyketide synthases
LAAO: L-amino acid oxidases
FFAs: Free fatty acids
TEM: Transmission electron microscope
PCA: Principal component analysis
ML: Maximum likelihood method
