# Supplementary material for: Genomic insights into the cellular specialization of predation in raptorial protists
Source: BMC Biol. 2024 May 7;22:107. doi: 10.1186/s12915-024-01904-2 (PMC11077807; doi:10.1186/s12915-024-01904-2)
Supplement: Supplementary file 1 — Additional file 1: Table S1. Differential Gene Statistics of Orthogroups in Haptoria, Trichostomatia and Spirotrichea. Gene counts of orthogroups in eight expanded gene families among Haptoria, Trichostomatia and Spirotrichea. Did.sp., Dilep.sp., Lacry.sp.1, Lacry.sp.2, Mono.sp., Trach.spp. from Haptoria, Ostra.dentatum, Dip.dentatum, Ento.caudatum, Epi.cattanei from Trichostomatia and Oxy.trifallax, Sty.lemnae from Spirotrichea. Table S2. DE genes associated with TCDB, PHI, VFDB in Haptoria, Trichostomatia and three Outgroup species. Differentially expressed gene about membrane transport proteins and cellular toxins. Lit.sp., Dil.mucronatus, Mon.sp., Did.sp., Tra.ovum, Phi.sp from Haptoria, Dip.dentatum, Ento.furca, Tri.finlayi, Bal.ctenopharyngodoni, Iso.intestinalis from Trichostomatia and Fab.salina, Tetra.thermophila, Param.bursaria as Outgroup species. Table S3. The source of ciliates genome and transcriptome data. We demonstrate the sources of all 62 genomic and transcriptomic datasets utilized in this study. Fig. S1. The distribution of gene length in litostomatea. Haptoria, Trichostomatia and Mesodinium, representing three types in litostomatea are all included. [file 12915_2024_1904_MOESM1_ESM.docx]

**Table S1. Differential Gene Statistics of Orthogroups in Haptoria, Trichostomatia and Spirotrichea**

| **Orthogroup id** | **Description** | **Did.sp.** | **Dilep.sp.** | **Lacry.sp.1** | **Lacry.sp.2** | **Mono.sp.** | **Trach.spp.** | **Ostra.dentatum** | **Dip.dentatum** | **Ento.caudatum** | **Epi.cattanei** | **Oxy.trifallax** | **Sty.lemnae** | **Total** |
| --- | --- | --- | --- | --- | --- | --- | --- | --- | --- | --- | --- | --- | --- | --- |
| OG0000604 | Ketoacyl-synt_C | 7 | 1 | 7 | 2 | 2 | 12 | 0 | 1 | 0 | 0 | 1 | 1 | 34 |
| OG0000690 | Thiolase_C | 4 | 2 | 3 | 1 | 5 | 14 | 0 | 1 | 0 | 0 | 1 | 1 | 32 |
| OG0003360 | DUF3050 | 1 | 0 | 3 | 4 | 1 | 1 | 0 | 0 | 0 | 0 | 0 | 1 | 11 |
| OG0000345 | LCCL | 8 | 11 | 10 | 10 | 3 | 4 | 0 | 0 | 0 | 0 | 0 | 0 | 46 |
| OG0001149 | LCCL | 4 | 8 | 6 | 5 | 0 | 0 | 0 | 0 | 0 | 0 | 0 | 0 | 23 |
| OG0001713 | LCCL | 4 | 6 | 5 | 2 | 1 | 0 | 0 | 0 | 0 | 0 | 0 | 0 | 18 |
| OG0004716 | LCCL | 3 | 0 | 1 | 1 | 1 | 2 | 0 | 0 | 0 | 0 | 0 | 0 | 8 |
| OG0000252 | TSP_1 | 14 | 1 | 14 | 10 | 6 | 8 | 0 | 0 | 0 | 0 | 0 | 0 | 53 |
| OG0003617 | TSP_1 | 4 | 3 | 0 | 1 | 1 | 1 | 0 | 0 | 0 | 0 | 0 | 0 | 10 |
| OG0005710 | TSP_1 | 1 | 2 | 1 | 1 | 1 | 1 | 0 | 0 | 0 | 0 | 0 | 0 | 7 |
| OG0006829 | COX1 | 0 | 1 | 3 | 2 | 0 | 0 | 0 | 0 | 0 | 0 | 0 | 0 | 6 |
| OG0009227 | COX1 | 1 | 0 | 1 | 1 | 0 | 0 | 0 | 0 | 0 | 0 | 0 | 1 | 4 |
| OG0002467 | DUF2451 | 2 | 1 | 2 | 5 | 3 | 1 | 0 | 0 | 0 | 0 | 0 | 0 | 14 |
| OG0001849 | Fibrinogen_C | 2 | 2 | 6 | 6 | 0 | 1 | 0 | 0 | 0 | 0 | 0 | 0 | 17 |
| OG0002220 | Proton_antipo_M | 4 | 3 | 3 | 3 | 1 | 1 | 0 | 0 | 0 | 0 | 0 | 0 | 15 |

**Table S2. DE genes associated with TCDB, PHI, VFDB in Haptoria, Trichostomatia and three Outgroup species**

|  | **Lit.sp.** | **Dil.mucronatus** | | **Mon.sp.** | **Did.sp.** | **Tra.ovum** | **Phi.sp.** | **Dip.dentatum** | **Ento.furca** | **Tri.finlayi** | **Bal.ctenopharyngodoni** | **Iso.intestinalis** | **Fab. salina** | **Tetra.thermophila** | **Param.bursaria** | **P value** | **FoldChange** |
| --- | --- | --- | --- | --- | --- | --- | --- | --- | --- | --- | --- | --- | --- | --- | --- | --- | --- |
| **TCDB-id** |  |  | |  |  |  |  |  |  |  |  |  |  |  |  |  |  |
| 1.A.105.1.2 | 43.86 | 0 | | 15.74 | 8.99 | 47.33 | 63.24 | 0 | 0 | 0 | 2.52 | 0 | 3.18 | 28.94 | 4.01 | 0.037 | 4.830 |
| 1.A.17.3.8 | 0 | 24.89 | | 17.18 | 44.08 | 30.38 | 18.21 | 0 | 0 | 0 | 0 | 0 | 0 | 0 | 50.33 | 0.014 | 7.811 |
| 1.A.3.2.3 | 45.73 | | 61.1 | 26.25 | 28.48 | 89.51 | 86.72 | 7.31 | 1.89 | 0 | 49.25 | 20.77 | 0 | 14.19 | 1.05 | 0.012 | 2.081 |
| 1.A.77.1.13 | 51.77 | | 491.38 | 234.12 | 184.94 | 194.56 | 649.24 | 11.83 | 6.84 | 6.78 | 122.97 | 62.49 | 2.09 | 6.39 | 82.41 | 0.032 | 3.080 |
| 2.A.1.53.4 | 3.23 | | 42.96 | 27.1 | 10.47 | 8.27 | 34.17 | 2.09 | 0 | 0 | 0 | 0 | 0 | 0 | 39.95 | 0.025 | 5.552 |
| 2.A.29.8.12 | 22.27 | | 27.38 | 41.06 | 24.35 | 89.07 | 52.87 | 0 | 0 | 0 | 0 | 0 | 23.13 | 8.49 | 5.59 | 0.011 | 3.437 |
| 2.A.53.3.8 | 106.83 | | 39.73 | 29.34 | 30.28 | 0 | 87.18 | 0 | 0 | 0 | 0 | 1.46 | 0 | 26.96 | 54.42 | 0.031 | 7.154 |
| 2.A.71.2.2 | 33.1 | | 13.13 | 34.08 | 67.47 | 99.28 | 95.2 | 5.31 | 9.8 | 0 | 6.42 | 13.6 | 0 | 0 | 52.21 | 0.017 | 3.260 |
| 3.A.1.204.6 | 0 | | 29.21 | 37.51 | 59.48 | 27.25 | 55.56 | 0 | 0 | 0 | 4.09 | 0 | 0 | 63.11 | 575.93 | 0.012 | 5.478 |
| 3.A.10.1.18 | 25.7 | | 298.67 | 737 | 605.93 | 236.63 | 456.26 | 0 | 0 | 0 | 0 | 55.15 | 10.74 | 208.91 | 498.51 | 0.015 | 5.150 |
| 3.D.3.1.2 | 0 | | 6.27 | 148.88 | 63.95 | 131.44 | 86.57 | 0 | 6.68 | 0 | 0 | 0 | 0 | 0 | 0 | 0.036 | 5.908 |
| 3.D.3.3.1 | 58.4 | | 60.38 | 137.53 | 54.34 | 152.01 | 365.06 | 0.94 | 0 | 0 | 0 | 69.1 | 0 | 61.89 | 266.82 | 0.048 | 3.551 |
| 8.A.114.1.6 | 384.7 | | 229.51 | 19.05 | 157.74 | 364.97 | 97.98 | 6.03 | 6.94 | 0 | 18.16 | 55.35 | 47.91 | 31.51 | 69.23 | 0.025 | 3.216 |
| 8.A.151.1.1 | 271.13 | | 466.74 | 1069.4 | 664.55 | 1399.81 | 1904.12 | 28.13 | 3.74 | 64 | 103.93 | 10.44 | 331.22 | 92.97 | 176.2 | 0.017 | 3.413 |
| **PHI-id** |  | |  |  |  |  |  |  |  |  |  |  |  |  |  |  |  |
| B2SI02 | 4966.73 | | 10762.2 | 0 | 4326.9 | 1102.42 | 7587.8 | 69.91 | 1333.09 | 918.8 | 45.65 | 7.19 | 0 | 1398.99 | 13827.9 | 0.043 | 3.597 |
| **VFDB-id** |  | |  |  |  |  |  |  |  |  |  |  |  |  |  |  |  |
| VFG001404 | 318.66 | | 227.97 | 258.09 | 156.68 | 412 | 0 | 20.89 | 0 | 0 | 2.52 | 0 | 0 | 56.16 | 17.73 | 0.011 | 5.838 |
| VFG005373 | 0 | | 25.71 | 12.72 | 110.27 | 74.67 | 67.55 | 0 | 0 | 0 | 0 | 0 | 0 | 34.85 | 0 | 0.038 | 8.921 |
| VFG016506 | 33.13 | | 6.38 | 72.63 | 24.27 | 54.84 | 0 | 0 | 0 | 0 | 0 | 2.98 | 0 | 60.71 | 63.42 | 0.040 | 5.739 |
| VFG044254 | 33.27 | | 56.82 | 191.12 | 36.49 | 114.27 | 60.58 | 25.03 | 0 | 0 | 5.22 | 13.48 | 12.2 | 34.14 | 13.02 | 0.032 | 3.123 |

**Table S3. The source of ciliates genome and transcriptome data**

| **Species** | **Genome** | **Transcriptome** | **Website** |
| --- | --- | --- | --- |
| *Lacrymaria* sp.1 | PRJCA019558 |  | https://ngdc.cncb.ac.cn/ |
| *Lacrymaria* sp.2 | PRJCA019558 |  | https://ngdc.cncb.ac.cn/ |
| Trachelophyllidae spp. | PRJCA019558 |  | https://ngdc.cncb.ac.cn/ |
| *Didinium* sp.1 | PRJCA019558 |  | https://ngdc.cncb.ac.cn/ |
| *Litonotus* sp.1 | PRJCA019558 |  | https://ngdc.cncb.ac.cn/ |
| *Dileptus* sp*.* | PRJCA019558 |  | https://ngdc.cncb.ac.cn/ |
| *Monodinium* sp. | PRJCA019558 | PRJNA1012847 | https://ngdc.cncb.ac.cn/  https://www.ncbi.nlm.nih.gov/ |
| *Didinium* sp.2 |  | PRJNA1012847 | https://www.ncbi.nlm.nih.gov/ |
| *Trachelius ovum* |  | PRJNA1012847 | https://www.ncbi.nlm.nih.gov/ |
| *Phialina* sp. |  | PRJNA1012847 | https://www.ncbi.nlm.nih.gov/ |
| *Litonotus* sp.2 |  | SRR10512988 | https://www.ncbi.nlm.nih.gov/ |
| *Dileptus mucronatus* |  | SRR10512990/SRR10512991 | https://www.ncbi.nlm.nih.gov/ |
| *Fabrea salina* |  | PRJCA019558 | https://www.ncbi.nlm.nih.gov/ |
| *Epidinium cattanei* | ASM2380562v1 |  | https://www.ncbi.nlm.nih.gov/ |
| *Epidinium caudatum* | ASM2380722v1 |  | https://www.ncbi.nlm.nih.gov/ |
| *Ostracodinium gracile* | ASM2380568v1 |  | https://www.ncbi.nlm.nih.gov/ |
| *Polyplastron multivesiculatum* | ASM2378335v1 |  | https://www.ncbi.nlm.nih.gov/ |
| *Diplodinium dentatum* | ASM2380716v1 | SRR8920957 | https://www.ncbi.nlm.nih.gov/ |
| *Diplodinium flabellum* | ASM2380556v1 |  | https://www.ncbi.nlm.nih.gov/ |
| *Entodinium bursa* | ASM2380734v1 |  | https://www.ncbi.nlm.nih.gov/ |
| *Entodinium caudatum* | ASM208785v3 |  | https://www.ncbi.nlm.nih.gov/ |
| *Isotricha intestinalis* | ASM2380706v1 | SRR8920958 | https://www.ncbi.nlm.nih.gov/ |
| *Isotricha* sp*.* | ASM2380574v1 |  | https://www.ncbi.nlm.nih.gov/ |
| *Isotricha prostoma* | ASM2380720v1 |  | https://www.ncbi.nlm.nih.gov/ |
| *Entodinium furca* |  | SRR8920956 | https://www.ncbi.nlm.nih.gov/ |
| *Trimyema finlayi* |  | SRR10356064 | https://www.ncbi.nlm.nih.gov/ |
| *Balantidium ctenopharyngodoni* |  | SRR5896119 | https://www.ncbi.nlm.nih.gov/ |
| *Mesodinium chamaeleon* |  | SRR9987803/SRR9987804 | https://www.ncbi.nlm.nih.gov/ |
| *Myrionecta rubra CCMP2563* |  | SRR1296700 | https://www.ncbi.nlm.nih.gov/ |
| *Mesodinium rubrum* |  | SRR2060969 | https://www.ncbi.nlm.nih.gov/ |
| *Climacostomum* sp. |  | [SRR10512972](https://trace.ncbi.nlm.nih.gov/Traces?run=SRR10512972) | https://www.ncbi.nlm.nih.gov/ |
| *Spirostomum* sp. |  | [SRR7141210](https://trace.ncbi.nlm.nih.gov/Traces?run=SRR7141210) | https://www.ncbi.nlm.nih.gov/ |
| *Stentor coeruleus* | GCA_001970955.1 |  | https://www.ncbi.nlm.nih.gov/ |
| *Climacostomum virens* |  | [SRR1300461](https://trace.ncbi.nlm.nih.gov/Traces?run=SRR1300461) | https://www.ncbi.nlm.nih.gov/ |
| *Blepharisma japonicum* | ERR6474356 | SRR1294460 | https://www.ncbi.nlm.nih.gov/ |
| *Loxodes striatus* |  | SRR10512997/SRR10512998 | https://www.ncbi.nlm.nih.gov/ |
| *Parduczia* sp. |  | SRR1300374 | https://www.ncbi.nlm.nih.gov/ |
| *Tetrahymena malaccensis* | GCA_000231845.2 |  | https://www.ncbi.nlm.nih.gov/ |
| *Tetrahymena thermophila* | GCA_016584475.1 |  | https://www.ncbi.nlm.nih.gov/ |
| *Ichthyophthirius multifiliis* | GCA_000220395.1 |  | https://www.ncbi.nlm.nih.gov/ |
| *Miamiensis avidus* | SRR15925892 |  | https://www.ncbi.nlm.nih.gov/ |
| *Pseudocohnilembus persalinus* | GCA_001447515.1 |  | https://www.ncbi.nlm.nih.gov/ |
| *Paramecium biaurelia* | GCA_000733385.1 |  | https://www.ncbi.nlm.nih.gov/ |
| *Paramecium tetraurelia* | GCA_000165425.1 |  | https://www.ncbi.nlm.nih.gov/ |
| *Frontonia* sp*.* |  | SRR19610302/SRR10512984 | https://www.ncbi.nlm.nih.gov/ |
| *Aristerostoma ATCC* |  | SRR1296811 | https://www.ncbi.nlm.nih.gov/ |
| *Bursaria truncatella* | SRR11061013 |  | https://www.ncbi.nlm.nih.gov/ |
| *Platyophrya macrostoma* |  | SRR1296905 | https://www.ncbi.nlm.nih.gov/ |
| *Dysteria derouxi* | SRR9841578 | SRR9841576 | https://www.ncbi.nlm.nih.gov/ |
| *Trochilia petrani* | SRR9841580 |  | https://www.ncbi.nlm.nih.gov/ |
| *Chilodochona* sp*.* | SRR9841583 |  | https://www.ncbi.nlm.nih.gov/ |
| *Favella ehrenbergii* |  | SRR1296812 | https://www.ncbi.nlm.nih.gov/ |
| *Schmidingerella taraikaensis* |  | SRR1296936/SRR1296899 | https://www.ncbi.nlm.nih.gov/ |
| *Strombidinopsis acuminatum* |  | SRR1296813 | https://www.ncbi.nlm.nih.gov/ |
| *Strombidium stylifer* | SRR8275879 |  | https://www.ncbi.nlm.nih.gov/ |
| *Oxytricha trifallax* | GCA_001297925.1 |  | https://www.ncbi.nlm.nih.gov/ |
| *Stylonychia lemnae* | GCA_000751175.1 |  | https://www.ncbi.nlm.nih.gov/ |
| *Halteria grandinella* | GCA_006369765.1 |  | https://www.ncbi.nlm.nih.gov/ |
| *Euplotes vannus* | Evan_data | | <https://evan.ciliate.org/index.php/home/downloads> |
| *Nyctotherus ovalis* |  | SRR10355986 | https://www.ncbi.nlm.nih.gov/ |
| *Brachonella spiralis* | SRR6033281 |  | https://www.ncbi.nlm.nih.gov/ |
| *Metopus* sp. |  | SRR10513011 | https://www.ncbi.nlm.nih.gov/ |


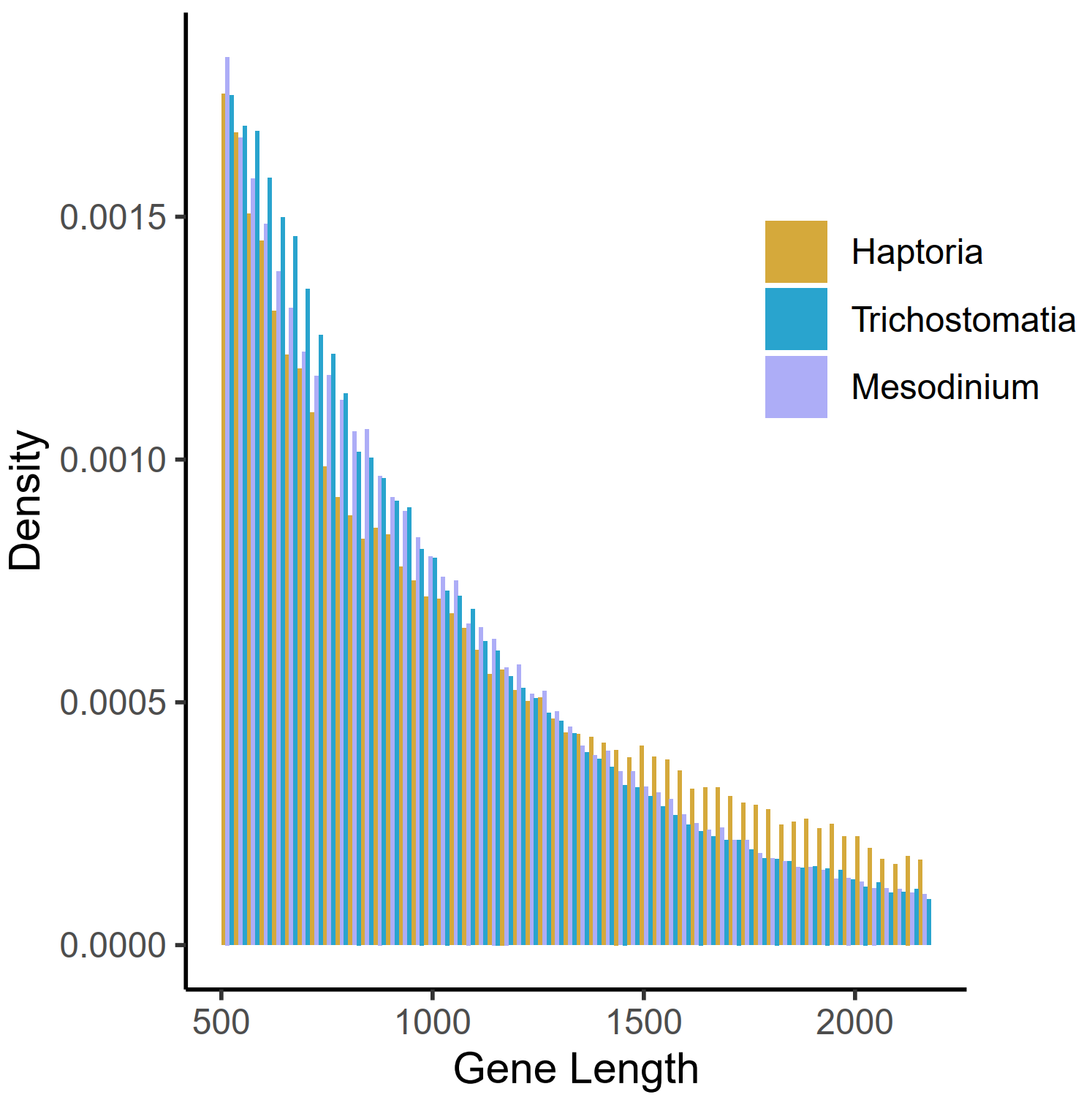


**Fig. S1 the distribution of gene length in litostomatea (including Haptoria, Trichostomatia and Mesodinium)**
